# Predictive Value of Varicocele Grade and Histopathology in Simultaneous Varicocelectomy and Sperm Retrieval in Non-Obstructive Azoospermia: A Retrospective Cohort Study

**DOI:** 10.3390/medicina60122056

**Published:** 2024-12-13

**Authors:** Aris Kaltsas, Fotios Dimitriadis, Michael Chrisofos, Nikolaos Sofikitis, Athanasios Zachariou

**Affiliations:** 1 Third Department of Urology, Attikon University Hospital, School of Medicine, National and Kapodistrian University of Athens, 12462 Athens, Greece; ares-kaltsas@hotmail.com (A.K.); mxchris@yahoo.com (M.C.); 2 Laboratory of Spermatology, Department of Urology, Faculty of Medicine, School of Health Sciences, University of Ioannina, 45110 Ioannina, Greece; nsofikit@uoi.gr; 3 Department of Urology, Faculty of Medicine, School of Health Sciences, Aristotle University of Thessaloniki, 54124 Thessaloniki, Greece; difotios@auth.gr

**Keywords:** testicular sperm extraction, varicocele grade, testicular histopathology, non-obstructive azoospermia, assisted reproductive technology

## Abstract

*Background and Objectives:* Varicocele repair in men with non-obstructive azoospermia (NOA) remains a subject of debate due to inconsistent outcomes. This study aimed to evaluate the impact of microsurgical varicocelectomy on sperm recovery rates in men with NOA and to assess the role of varicocele grade and testicular histopathology in predicting postoperative outcomes. *Materials and Methods:* A retrospective cohort study was conducted of 78 men diagnosed with NOA and clinical varicocele who underwent microsurgical subinguinal varicocelectomy with simultaneous diagnostic and therapeutic testicular biopsy at the Department of Urology of the University of Ioannina between September 2013 and December 2021. Varicoceles were graded I to III based on physical examination and Doppler ultrasound. Histopathological patterns were classified as hypospermatogenesis (HYPO), early maturation arrest (EMA), late maturation arrest (LMA), or Sertoli cell-only syndrome (SCOS). Patients were followed postoperatively at 3, 6, 9, and 12 months, with semen analyses performed according to World Health Organization guidelines to assess sperm presence. *Results:* At the 12-month follow-up, spermatozoa were detected in the ejaculate of 26 out of 78 patients, resulting in an overall sperm return to ejaculate rate of 33.3%. Varicocele grade significantly influenced outcomes: patients with Grade II varicoceles had the highest sperm return to ejaculate rate (45.2%, 14/31), followed by Grade III (39.1%, 9/23) and Grade I (12.5%, 3/24) (*p*-value < 0.05). The sperm retrieval rate (SRR) from testicular biopsies also varied with varicocele grade: Grade II had the highest SRR (54.8%, 17/31), followed by Grade III (47.8%, 11/23) and Grade I (33.3%, 8/24). Histopathological findings were significant predictors of sperm retrieval: patients with HYPO had an SRR of 84.8% (28/33) and a sperm return to ejaculate rate of 66.7% (22/33); those with LMA had an SRR of 47.1% (8/17) and a sperm return rate of 23.5% (4/17). No sperm retrieval was observed in patients with EMA (0%, 0/4) or SCOS (0%, 0/24) (*p*-value < 0.01). Multivariate logistic regression identified varicocele grade and histopathology as independent predictors of sperm retrieval, with higher grades and favorable histopathology associated with increased likelihood of success. *Conclusions:* Microsurgical varicocelectomy can induce spermatogenesis in a significant proportion of men with NOA, particularly those with higher-grade varicoceles and favorable histopathological patterns such as HYPO or LMA. Varicocele grade and histopathological findings are important predictors of postoperative outcomes and should inform patient selection and counseling. These findings provide valuable insights for optimizing fertility treatments in men with NOA undergoing varicocele repair.

## 1. Introduction

Varicocele, an abnormal dilation of the pampiniform venous plexus within the scrotum, is a common cause of male infertility and is frequently associated with non-obstructive azoospermia (NOA) [1]. NOA, characterized by impaired spermatogenesis resulting in a complete absence of sperm in the ejaculate, is the most severe form of male infertility, affecting approximately 1% of all men and 10–15% of infertile men [2]. While varicocele is often linked to testicular dysfunction and decreased semen quality, its exact role in the pathophysiology of NOA remains a subject of ongoing debate [3,4].

The relationship between varicocele and NOA is complex and not fully understood [5]. Proposed mechanisms by which varicoceles may impair spermatogenesis include increased scrotal temperature due to impaired venous drainage, hypoxia from altered testicular blood flow, oxidative stress caused by the accumulation of reactive oxygen species, and hormonal imbalances affecting the hypothalamic-pituitary-gonadal axis [6]. Elevated testicular temperature can adversely affect Leydig and Sertoli cell function, leading to decreased testosterone production and disrupted support for germ cell development [7,8]. Hypoxia resulting from venous stasis may further exacerbate testicular damage, and increased ROS levels can damage sperm DNA and cellular structures [9]. These factors collectively contribute to testicular dysfunction and impair the process of sperm production.

Varicocelectomy, the surgical repair of varicoceles, has been proposed as a treatment to restore spermatogenesis in men with NOA [10]. The procedure aims to alleviate the detrimental effects of varicoceles by correcting venous reflux. Restoration of normal testicular temperature can enhance Leydig cell function, leading to increased testosterone levels essential for spermatogenesis [11]. Improvement in Sertoli cell function may also occur, promoting the progression of germ cells through meiosis and spermiogenesis [12]. By mitigating oxidative stress and enhancing the testicular microenvironment, varicocelectomy may convert an azoospermic phenotype to an oligospermic one in some patients [13].

However, studies investigating its effectiveness have yielded mixed results [4,14]. Some research indicates that varicocelectomy can induce spermatogenesis and improve sperm retrieval rates, leading to the reappearance of sperm in the ejaculate and enhancing the potential for natural conception or use in assisted reproductive technologies (ART) [15]. Conversely, other studies report limited or inconsistent benefits, particularly in patients with severe testicular damage or unfavorable histopathological patterns, such as Sertoli cell-only syndrome (SCOS) [16].

The variability in outcomes following varicocelectomy may be attributed to factors such as varicocele grade, testicular histopathology, hormonal profiles, and differences in patient selection criteria [17]. Despite advancements in ART, notably intracytoplasmic sperm injection (ICSI), which allow for the use of even a few viable sperm, optimizing pre-ART interventions like varicocele repair remains critical [18,19]. Currently, there is no consensus on which patients with NOA are most likely to benefit from varicocelectomy, and the procedure’s true clinical utility continues to be debated [20].

A significant gap exists in the literature regarding the comprehensive evaluation of how varicocele grade and testicular histopathology impact the outcomes of varicocele repair in men with NOA [21]. Most existing studies lack detailed analyses correlating these factors with postoperative sperm recovery, which is crucial for identifying patients who may derive the most benefit from surgery [22]. Addressing this gap is essential for developing personalized treatment strategies and improving fertility outcomes in this challenging patient population.

The aim of this retrospective cohort study is to assess the effectiveness of microsurgical varicocelectomy in inducing spermatogenesis in men with NOA and to determine how varicocele grade and histopathological findings influence postoperative sperm retrieval rates and the return of sperm to the ejaculate. By analyzing these variables, we seek to identify predictors of successful sperm recovery, thereby providing evidence to guide clinical decision-making and optimize patient selection for varicocelectomy.

## 2. Materials and Methods Study Design

A retrospective cohort study was conducted at the Department of Urology of the University of Ioannina from September 2013 to December 2021. The primary objective was to evaluate the impact of microsurgical varicocele repair on spermatogenesis in men with NOA. Postoperative sperm recovery was assessed through semen analysis and correlation with histopathological findings from diagnostic testicular biopsies (DTB). Follow-up evaluations were conducted at 3, 6, 9, and 12 months postoperatively.

### 2.1. Ethical Considerations

The study protocol received approval from the departmental ethics committee and adhered to the principles of the Declaration of Helsinki. Patient data were anonymized to ensure confidentiality, and identifiable information was removed or de-identified before analysis.

### 2.2. Patient Selection

Seventy-eight men diagnosed with NOA and clinical varicocele were included. Clinical assessment included bilateral palpation of the vas deferens, detailed medical and reproductive histories, physical examinations, semen analysis, and endocrinological evaluations. Varicoceles were graded using a standard classification system [23]: Grade I, palpable only during the Valsalva maneuver; Grade II, palpable at rest; and Grade III, both visible and palpable at rest. Scrotal color Doppler ultrasound was used for confirmation but did not influence grading. Subclinical varicoceles detectable only via ultrasound were excluded. Additional parameters included age, testicular volume (measured via scrotal ultrasound), laterality (left-sided or bilateral varicoceles), and presence of right-sided varicocele.

Inclusion criteria required patients to be male, aged 18 years or older, and with a confirmed diagnosis of azoospermia based on at least two semen analyses conducted one month apart, following World Health Organization (WHO) guidelines [24]. Azoospermia was confirmed by centrifugation of semen samples at 3000× *g* for 15 min to detect any sperm presence. Patients were also required to have a normal ejaculate volume (>1.5 mL) and an absence of obstructive causes of azoospermia, confirmed by physical examination (e.g., the presence of bilateral vas deferens and epididymis). Inclusion criteria also specified that patients should have no prior varicocele repair or other surgical interventions affecting fertility. Preoperative hormonal assessments were conducted to measure levels of follicle-stimulating hormone (FSH), luteinizing hormone (LH), and total testosterone (TT).

Patients were excluded from the study if they had evidence of genetic abnormalities identified through karyotype analysis and Y-chromosome microdeletion testing (such as Klinefelter syndrome or AZF region deletions) [20], significant hormonal imbalances (e.g., hyperprolactinemia), previous chemotherapy or radiotherapy, a history of obstructive azoospermia, prior surgeries affecting fertility (e.g., vasectomy), or comorbid conditions known to impair fertility (e.g., uncontrolled diabetes mellitus). Additionally, systemic congenital diseases such as sickle cell disease and patients with a history of malignancy, which may affect testicular function, were also excluded to ensure a more homogenous study population. Klinefelter syndrome, though not a contraindication for mTESE or varicocele repair, was excluded in this study to maintain consistency in baseline characteristics. Men with Klinefelter syndrome frequently present with significant endocrine alterations and testicular histology that could independently affect outcomes. The exclusion ensures the focus remains on evaluating varicocele grade and histopathological findings in predicting postoperative outcomes.

### 2.3. Surgical Procedure

Patients underwent microsurgical subinguinal varicocelectomy combined with DTB and therapeutic testicular biopsy (TTB) using microdissection testicular sperm extraction (mTESE). All procedures were performed simultaneously under general anesthesia by an experienced microsurgeon. By combining microsurgical varicocelectomy with DTB and TTB, the approach aimed to alleviate the detrimental effects of varicocele on spermatogenesis by correcting venous reflux and improving testicular microcirculation. DTB allowed for the evaluation of testicular histopathology to determine the underlying cause of azoospermia, exclude malignancy, and guide prognosis. TTB with mTESE provided an opportunity to obtain sperm directly from the testicular tissue for use in ART, increasing the chances of achieving biological paternity.

### 2.4. Histopathological Analysis

**Figure 1:**
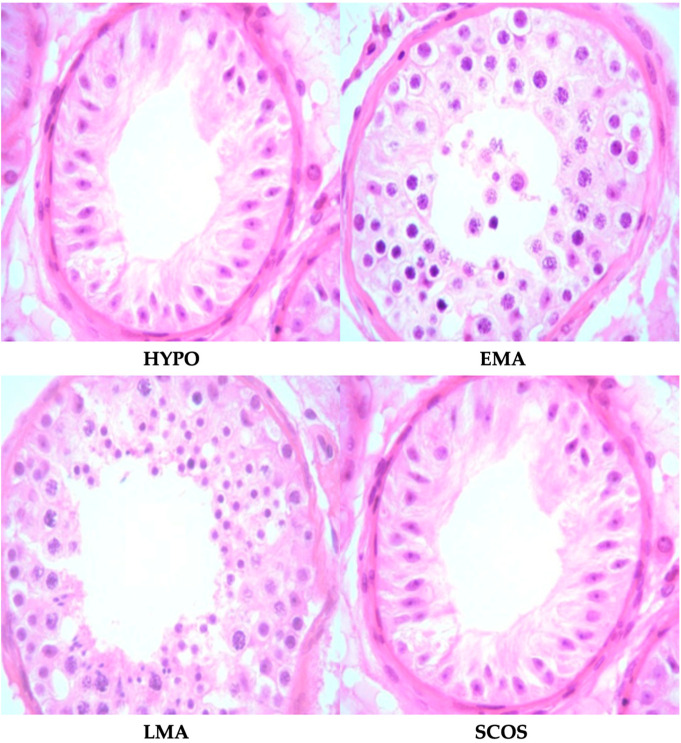
Histopathological patterns of testicular biopsies.

Testicular biopsy specimens were fixed in Bouin’s solution (Merck KGaA, Darmstadt, Germany), embedded in paraffin, sectioned, and stained with hematoxylin and eosin (Merck KGaA, Darmstadt, Germany). A single experienced pathologist, blinded to clinical data, evaluated the slides. Histopathological patterns were classified into four categories [25]: hypospermatogenesis (HYPO), early maturation arrest (EMA), late maturation arrest (LMA), and SCOS. These classifications reflect the state of spermatogenesis and are critical for predicting outcomes in fertility treatments. The histopathological patterns are illustrated in Figure 1 (Scale bar: 100 µm, Magnification: ×40).

HYPO represents a reduced but still active process of spermatogenesis. Maturation arrest, EMA and LMA stages, occurs when spermatogenesis halts at specific developmental stages. In EMA, the process stops early, typically at the spermatocyte stage, meaning no mature sperm cells are produced. In contrast, LMA involves a halt at the spermatid stage, where sperm are closer to full maturation. SCOS is a severe condition where the seminiferous tubules contain only Sertoli cells, without any germ cells necessary for sperm production [26]. These patterns provide prognostic information crucial for predicting the likelihood of successful sperm retrieval [27].

### 2.5. Postoperative Follow-Up and Semen Analysis

Follow-up visits occurred at 3, 6, 9, and 12 months postoperatively. Semen samples were collected by masturbation after 2–5 days of sexual abstinence and analyzed according to WHO 2010 criteria [24]. Parameters included sperm concentration, motility, and morphology.

The primary outcome was the presence of spermatozoa in the ejaculate post-surgery, categorized as:

- Persistent: Spermatozoa present at all follow-up points.
- Intermittent: Spermatozoa present at some follow-up points but absent at others
- Relapse: Initial sperm recovery followed by a return to azoospermia.

### 2.6. Hormonal Analysis

Preoperative serum levels of FSH, LH, and testosterone were measured using chemiluminescent immunoassays. These hormonal parameters were analyzed for correlations with postoperative sperm recovery, providing insights into potential predictors of successful spermatogenesis following varicocele repair.

### 2.7. Statistical Analysis

Statistical analyses were conducted using SPSS software version 22.0 (IBM Corp., Armonk, NY, USA). Baseline characteristics were summarized using mean ± standard deviation (SD) for normally distributed continuous variables, median and interquartile range (IQR) for non-normally distributed variables, and frequencies and percentages for categorical variables.

The Shapiro–Wilk test assessed the normality of continuous variables. Due to the non-normal distribution of sperm counts (preoperative counts were zero; postoperative counts were skewed with many zeros), non-parametric tests were utilized.

### 2.8. Comparative Analyses

Given that all patients were azoospermic preoperatively, and postoperative sperm presence represented a clinically significant change, the increase in sperm presence was presented descriptively without formal statistical testing. This approach was chosen because statistical tests comparing preoperative and postoperative sperm presence are not meaningful when the preoperative variable has no variation (all zeros).

The Mann–Whitney U test was employed to compare continuous variables between patients who achieved sperm retrieval and those who did not, due to the non-normal distribution of data. The chi-square test or Fisher’s exact test was applied for the comparison of categorical variables, such as sperm retrieval rates and histopathological patterns, between groups.

### 2.9. Correlation Analyses

Spearman’s rank correlation coefficient (rho) evaluated the relationship between sperm retrieval and variables like age, hormonal levels, varicocele grade, and histopathological findings.

### 2.10. Multivariate Logistic Regression Analysis

To identify independent predictors of postoperative sperm retrieval, multivariate logistic regression was performed. Variables included were those with a *p*-value < 0.10 in univariate analyses and clinically relevant factors:

- Varicocele grade (I, II, III)
- Histopathological patterns (SCOS, EMA, LMA, HYPO)
- Age
- Preoperative hormonal levels (FSH, LH, testosterone)
- Bilateral varicocele (Yes vs. No)

Odds ratios (OR) with 95% confidence intervals (CI) were calculated for each variable. Model fit was assessed using the Hosmer–Lemeshow goodness-of-fit test. Multicollinearity was evaluated using variance inflation factors (VIF), with VIF >10 indicating significant multicollinearity.

### 2.11. Significance Level

A two-tailed *p*-value of less than 0.05 was considered statistically significant for all tests.

## 3. Results

### 3.1. Patient Demographics and Baseline Characteristics

A total of 78 men with NOA and clinical varicocele were included in the study. The mean age of the patients was 33.9 ± 7.6 years. All patients had left-sided varicoceles, with 17 patients (21.8%) also presenting with bilateral varicoceles. Varicoceles were classified as Grade I in 24 patients (30.8%), Grade II in 31 patients (39.7%), and Grade III in 23 patients (29.5%). Preoperative hormone levels, including FSH, LH, and testosterone, were within expected ranges for this patient group. Detailed demographic and baseline characteristics are presented in Table 1.

### 3.2. Postoperative Sperm Return to Ejaculate

At the 12-month follow-up, spermatozoa were detected in the ejaculate of 26 out of 78 patients, resulting in an overall sperm return-to-ejaculate rate of 33.3%. Among these 26 patients, the consistency of sperm presence was categorized as follows:

- Persistent sperm presence: 16 patients (61.5%) had spermatozoa detected at all postoperative follow-up points.
- Intermittent sperm recovery: five patients (19.2%) had spermatozoa present at some but not all follow-up points.
- Relapse to azoospermia: five patients (19.2%) initially showed sperm recovery but relapsed to azoospermia by the 12-month follow-up.

### 3.3. Effect of Varicocele Grade on Sperm Return to Ejaculate and Testicular Sperm Retrieval Rates

**Figure 2:**
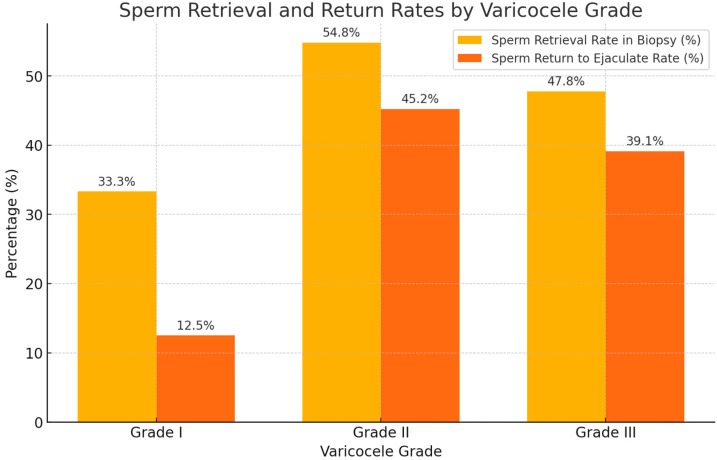
Sperm return to ejaculate and testicular sperm retrieval rates by varicocele grade.

Varicocele grade had a significant impact on both sperm return to the ejaculate and testicular sperm retrieval rates (SRR). Patients with Grade II varicoceles had a significantly higher sperm return to ejaculate rate (45.2%) compared to those with Grade I varicoceles (12.5%; *p* = 0.006, chi-square test). Similarly, the SRR from testicular biopsies was highest in Grade II patients (54.8%). These variations are visually depicted in Figure 2, which illustrates the comparative SRR and sperm return rates across varicocele grades, emphasizing the favorable outcomes associated with higher-grade varicoceles.

### 3.4. Kaplan–Meier Analysis of Time to Sperm Recovery

**Figure 3:**
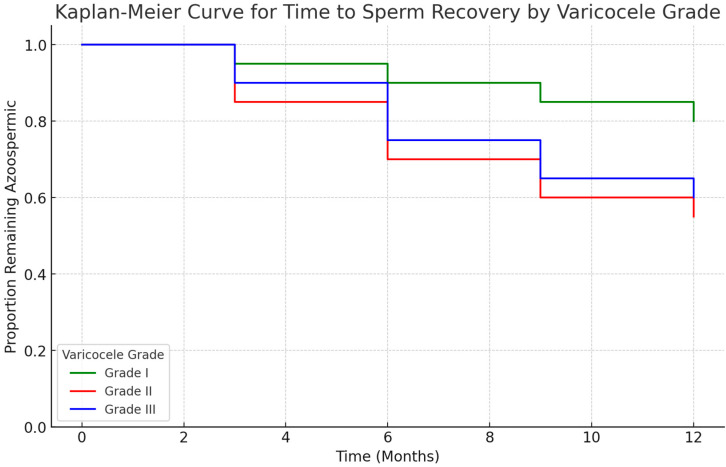
Kaplan–Meier curves illustrating the time to sperm recovery after microsurgical varicocelectomy in patients with non-obstructive azoospermia, stratified by varicocele grade.

The temporal dynamics of postoperative sperm recovery were evaluated using Kaplan–Meier survival analysis. Figure 3 illustrates the Kaplan–Meier curves for time to sperm recovery stratified by varicocele grade.

Patients with Grade II varicoceles demonstrated the fastest recovery, as evidenced by a steep decline in the proportion of patients with azoospermia during the first 6 months, postoperatively. By the 6-month follow-up, a significant number of patients with Grade II had achieved sperm recovery. In contrast, patients with Grade I varicoceles exhibited the slowest recovery rates, with a higher proportion remaining azoospermic throughout the 12-month follow-up period. The gradual decline in azoospermia among this group suggests limited immediate benefit from varicocelectomy. Patients with Grade III varicoceles showed intermediate recovery rates; while improvements were observed, the recovery was not as rapid or as extensive as in patients with Grade II.

### 3.5. Correlation with Histopathological Findings

The overall SRR from testicular biopsies was 46.2% (36 out of 78 patients). Significant variations were observed based on histopathological patterns. Patients with HYPO had the highest SRR, with 28 out of 33 patients (84.8%) having sperm detected in biopsies, and 22 patients (66.7%) experiencing sperm return to the ejaculate. For patients with LMA, 8 out of 17 patients (47.1%) had sperm detected in biopsies, and 4 patients (23.5%) had sperm return to the ejaculate.

In contrast, no sperm retrieval from either biopsies or ejaculate was observed in patients with EMA or SCOS; all 4 patients with EMA and all 24 patients with SCOS had a 0% sperm retrieval rate in both biopsy and ejaculate outcomes.

Statistical analysis using Fisher’s exact test revealed that patients with HYPO had a significantly higher SRR compared to those with SCOS (*p*-value < 0.001). Similarly, patients with LMA had a significantly higher SRR compared to SCOS (*p*-value < 0.001). Sperm return to ejaculate rates also differed significantly among histopathological patterns (*p*-value < 0.001). Table 2 summarizes the sperm retrieval rates according to histopathological patterns.

### 3.6. Correlation with Hormonal Levels and Other Clinical Variables

Spearman’s rank correlation coefficients revealed no significant correlations between sperm retrieval and preoperative hormonal levels: FSH (rho = −0.10, *p* = 0.35), LH (rho = −0.08, *p* = 0.45), and testosterone (rho = 0.12, *p* = 0.28). Additionally, no significant associations were identified between sperm retrieval and other factors, such as patient age (rho = 0.07, *p* = 0.56) or the presence of bilateral varicocele (rho = 0.03, *p* = 0.75).

### 3.7. Multivariate Logistic Regression Analysis

A multivariate logistic regression analysis was conducted to identify independent predictors of sperm retrieval from the testis. The model included varicocele grade, histopathological findings, age, preoperative hormonal levels (FSH, LH, TT), and the presence of bilateral varicocele. With 36 events (patients with successful sperm retrieval) and six predictor variables, the model met the recommended events-per-variable ratio for logistic regression.

Patients with Grade II varicocele had a significantly higher likelihood of sperm retrieval compared to those with Grade I varicocele (odds ratio [OR] = 5.87; 95% confidence interval [CI]: 1.48–23.28; *p* = 0.012). Grade III varicocele showed a non-significant trend toward increased sperm retrieval (OR = 4.35; 95% CI: 0.90–21.07; *p* = 0.067).

Histopathological findings were significant predictors: patients with HYPO had a higher likelihood of sperm retrieval compared to those with SCOS (OR = 8.25; 95% CI: 2.06–33.02; *p* = 0.003), and patients with LMA also had increased odds compared to SCOS (OR = 7.60; 95% CI: 1.08–53.48; *p* = 0.041). Other variables, including age, FSH, LH, testosterone levels, and bilateral varicocele, were not significant predictors (*p* > 0.05 for all).

The logistic regression model demonstrated good fit, as indicated by the Hosmer–Lemeshow goodness-of-fit test (χ^2^ = 4.32, df = 8, *p* = 0.827). The area under the receiver operating characteristic (ROC) curve was 0.85, indicating good discriminative ability of the model. Variance inflation factors (VIFs) for all variables were less than 2, suggesting no significant multicollinearity. Detailed results are shown in Table 3.

## 4. Discussion

This retrospective cohort study evaluated the effectiveness of simultaneous microsurgical varicocelectomy and testicular sperm retrieval in men with NOA and clinical varicocele. The primary aim was to determine how varicocele grade and testicular histopathology influence postoperative sperm retrieval rates from testicular biopsies and the return of sperm to the ejaculate. The findings demonstrate that varicocelectomy can induce spermatogenesis in a significant subset of men with NOA, particularly those with higher-grade varicoceles and favorable histopathological patterns such as HYPO or LMA. This aligns with prior studies, where varicocelectomy has been shown to improve sperm retrieval rates, especially in patients with favorable histopathology [28,29].

At the 12-month follow-up, spermatozoa were detected in the ejaculate of 33.3% of patients, indicating that varicocelectomy effectively enhanced spermatogenesis in these individuals. Additionally, the overall SRR from testicular biopsies performed during surgery was 46.2%, which is consistent with previous findings showing improvement in SRR post-varicocelectomy [30]. Notably, both varicocele grade and histopathological findings emerged as significant predictors of postoperative outcomes, emphasizing the importance of personalized treatment strategies.

### 4.1. Impact of Varicocele Grade on Sperm Retrieval and Spermatogenesis

Varicocele grade significantly influenced both the return of sperm to the ejaculate and the SRR from testicular biopsies. Patients with Grade II varicoceles exhibited the highest rates of sperm recovery, with a 45.2% return to ejaculate rate and a 54.8% SRR from biopsies. Grade III varicoceles had slightly lower rates, at 39.1% and 47.8%, respectively, while Grade I varicoceles showed the lowest rates, at 12.5% for both measures.

### 4.2. Temporal Patterns of Sperm Recovery

The Kaplan–Meier survival analysis provided valuable insights into the temporal patterns of sperm recovery post-varicocelectomy. Patients with Grade II varicoceles experienced the most rapid recovery of spermatogenesis, with a significant proportion regaining sperm in their ejaculate within the first 6 months. This swift improvement underscores the potential effectiveness of varicocelectomy in patients with moderate varicocele severity, where the balance between testicular damage and the capacity for recovery is optimal.

In contrast, patients with Grade I varicoceles exhibited the slowest and least pronounced recovery over time. The higher proportion of patients with azoospermia throughout the 12-month follow-up suggests that mild varicoceles may not significantly impair spermatogenesis or that other underlying factors contribute to azoospermia in these individuals. Consequently, the benefit of varicocelectomy in Grade I varicocele patients may be limited.

Grade III varicocele patients demonstrated intermediate recovery rates. While some improvement was noted, the extent and speed of recovery were less than those observed in Grade II patients. This may be due to more extensive testicular damage caused by severe varicoceles, which could require a longer time for recovery or may result in irreversible impairment. These differences in recovery times among varicocele grades were statistically significant, as indicated by the log-rank test (*p*-value = 0.015). This finding highlights the importance of varicocele grade not only in predicting the likelihood of postoperative sperm recovery but also in determining the timing of recovery.

### 4.3. Interpretation and Clinical Significance

The superior outcomes in Grade II varicoceles suggest that while these varicoceles cause significant impairment of testicular function due to venous reflux and scrotal hyperthermia, the damage remains reversible upon surgical intervention. The slightly lower recovery rates in Grade III varicoceles could be attributed to more extensive or irreversible testicular damage resulting from prolonged venous reflux [31]. Chronic exposure to elevated temperatures and oxidative stress in Grade III varicoceles may lead to degeneration of the seminiferous epithelium, thereby limiting the potential for spermatogenesis to resume even after varicocelectomy. These findings align with prior studies indicating that higher-grade varicoceles are associated with more pronounced testicular dysfunction but also offer greater potential for recovery when repaired surgically [32,33].

The limited success and slow recovery in Grade I varicocele cases underscore the importance of careful patient selection. Mild venous reflux may not be the primary cause of azoospermia in these patients, suggesting that other underlying factors, such as genetic or idiopathic testicular dysfunction, may contribute to infertility. Therefore, varicocelectomy may provide limited benefit in enhancing spermatogenesis for this group, and alternative treatment strategies should be considered [34].

### 4.4. Histopathological Findings

Histopathological analysis revealed that patients with HYPO had the highest rates of both sperm retrieval from testicular biopsies and return of sperm to the ejaculate, at 84.8% and 66.7%, respectively. This indicates that varicocelectomy effectively enhances spermatogenesis in patients where the spermatogenic process is reduced but still active. The high SRR during surgery and subsequent appearance of sperm in the ejaculate suggest that correcting the varicocele can restore sufficient testicular function in these individuals. These findings are consistent with research indicating that men with HYPO show the best outcomes following varicocele repair [35].

Patients with LMA showed moderate success, with a 47.1% SRR from biopsies and a 23.5% return to ejaculate rate. This reflects partial restoration of spermatogenesis, as these patients have spermatogenesis arrested at a later stage (spermatid stage), and varicocelectomy may help resume the maturation process. However, the lower rates compared to HYPO indicate that the extent of impairment in LMA limits the overall recovery potential. In contrast, patients with EMA and SCOS had 0% SRR from biopsies and no return of sperm to the ejaculate. These conditions represent severe spermatogenic failure, with either early arrest of germ cell development or complete absence of germ cells. The inability of varicocelectomy to enhance spermatogenesis in these patients suggests that factors other than venous reflux, such as intrinsic testicular pathology, are the primary causes of azoospermia [36].

These findings are consistent with previous studies that have identified histopathological patterns as critical predictors of sperm retrieval outcomes. Esteves et al. reported higher sperm retrieval rates in men with HYPO compared to those with maturation arrest or SCOS [30]. These results reinforce the importance of histopathological evaluation in guiding clinical decision-making and patient counseling.

Performing varicocelectomy and testicular sperm retrieval concurrently during the same surgical session offers several advantages. Firstly, it maximizes the chances of obtaining sperm for immediate use in ART, particularly for patients who may not have sperm return to their ejaculate postoperatively. Secondly, it minimizes the need for multiple surgical interventions, reducing patient burden and healthcare costs. In this study, nearly half of the patients had sperm successfully retrieved directly from testicular tissue during the procedure, providing immediate options for ART.

The combined approach is especially beneficial for patients with favorable prognostic factors, such as higher-grade varicoceles and HYPO histopathology. For patients with poor prognostic indicators, such as EMA or SCOS, the simultaneous procedure allows for definitive assessment and avoids unnecessary delays in pursuing alternative reproductive options.

The observed improvements in spermatogenesis following varicocelectomy can be attributed to several physiological mechanisms. Correction of venous reflux reduces intratesticular pressure and improves arterial inflow, enhancing testicular hemodynamics. This results in better oxygenation and nutrient delivery to the seminiferous tubules, which is essential for germ cell development. Additionally, reducing oxidative stress by decreasing ROS mitigates damage to spermatogenic cells. Varicocelectomy also normalizes testicular temperature, crucial for maintaining optimal conditions for sperm production. While hormonal levels were not predictive in this study, improved testicular function may enhance Leydig cell activity, contributing to a hormonal environment conducive to spermatogenesis [21].

### 4.5. Correlation with Hormonal Levels and Other Clinical Variables

The present study found no significant correlations between preoperative hormonal levels (FSH, LH, TT) and postoperative sperm retrieval outcomes. This suggests that while hormonal profiles reflect overall testicular function, they may not reliably predict the success of varicocelectomy in enhancing spermatogenesis. Similar findings have been reported in other studies, indicating that hormonal levels alone should not be used to determine the suitability of patients for varicocele repair [37].

Other clinical variables, such as patient age, testicular volume, and the presence of bilateral varicoceles, have also been considered potential factors influencing spermatogenesis. Advanced age may negatively impact testicular function and sperm quality due to physiological changes [38], and reduced testicular volume is often associated with diminished spermatogenic activity [39]. Bilateral varicoceles could exacerbate testicular dysfunction because of increased venous reflux and thermal stress [40]. This highlights that varicocele grade and histopathological findings are more critical determinants of postoperative outcomes, and these should be the primary considerations in patient selection and counseling [28,36].

### 4.6. Risk of Relapse and Importance of Sperm Cryopreservation

A notable finding in this study was that 19.2% of patients who initially showed sperm recovery experienced a relapse to azoospermia by the 12-month follow-up. This underscores the potential for transient improvements in spermatogenesis following varicocelectomy. Similar occurrences of relapse have been reported in other studies, indicating that sperm recovery may not be permanent for all patients [36].

The risk of relapse necessitates early sperm cryopreservation once spermatozoa are detected, either during surgery or in the ejaculate postoperatively. Cryopreservation ensures that viable sperm are available for future use in ART, safeguarding against the possibility of subsequent azoospermia and providing patients with continued reproductive options [14].

### 4.7. Clinical Implications

The findings of this study hold significant clinical implications for the management of men with NOA and clinical varicocele. The results indicate that varicocelectomy, particularly when combined with testicular sperm retrieval, may improve outcomes for patients with higher-grade varicoceles (Grade II and III). Such interventions can lead to increased chances of sperm retrieval and potentially the return of sperm to the ejaculate, which may facilitate natural conception or simplify ART protocols [35].

Histopathological patterns, such as HYPO and LMA, are associated with higher success rates in sperm retrieval but are identified through postoperative analysis and therefore cannot serve as preoperative clinical indications. Instead, preoperative counseling should rely on hormonal levels and varicocele grade, which are established as reliable prognostic indicators. Diagnostic biopsies, while performed intraoperatively, are invaluable for predicting fertility outcomes, as studies demonstrate a strong correlation between histological findings and later sperm retrieval success rates [41]. Postoperatively, these histopathological insights confirm treatment efficacy and guide future reproductive decisions, such as whether to pursue ART or alternative strategies.

For patients with lower-grade varicoceles or unfavorable histopathology (EMA or SCOS), varicocelectomy may offer limited benefit in enhancing spermatogenesis. In such cases, counseling should focus on alternative reproductive strategies, such as the use of donor sperm or adoption. The decision to proceed with surgery should be individualized, taking into account the patient’s reproductive goals, preferences, and the potential risks and benefits of the procedure [36]. By integrating these findings with preoperative clinical and hormonal assessments, clinicians can provide more precise counseling, manage patient expectations effectively, and facilitate shared decision-making tailored to individual reproductive goals.

### 4.8. Limitations

Several limitations inherent to this study may affect the interpretation of the findings. First, the retrospective design may have introduced selection bias, as inclusion was limited to patients who underwent simultaneous microsurgical varicocelectomy and testicular biopsy at a single institution between 2013 and 2021. Patients with better baseline characteristics or greater motivation to pursue surgical intervention might have been more likely to participate, potentially leading to an overestimation of success rates.

Second, over the eight-year study period, variations in surgical techniques, postoperative care, and laboratory assessments—including histopathological evaluations—could have introduced variability in the data. Although all surgeries were performed by an experienced microsurgeon, subtle differences in technique and practice over time might have influenced outcomes.

Third, the histopathological analyses were conducted by a single pathologist. While this approach ensures consistency in evaluation criteria, it may introduce observer bias and limit the reproducibility of the findings. Inter-observer variability is a known issue in testicular histopathology; involving multiple pathologists could have strengthened the validity of the classifications. Fourth, the study was conducted at a single tertiary care center with a relatively small sample size of 78 patients. When stratified by varicocele grade and histopathological patterns, some subgroups—particularly those with EMA and SCOS—had few patients (4 and 24 patients, respectively). This small number reduces the statistical power to detect significant differences and limits the generalizability of the results to a broader population.

Fifth, the absence of a control group of NOA patients with varicocele who did not undergo varicocelectomy makes it challenging to attribute improvements in spermatogenesis solely to the surgical intervention. Other factors, such as lifestyle changes or hormonal fluctuations, may have contributed to the observed outcomes. Finally, the follow-up period of 12 months may not be sufficient to assess long-term reproductive outcomes such as pregnancy and live birth rates. These endpoints are critical in fertility studies to determine the ultimate effectiveness of varicocelectomy in achieving biological paternity. Future studies with longer follow-up periods and inclusion of reproductive outcomes are necessary to fully evaluate the clinical benefits of the procedure.

### 4.9. Future Directions

Future research should aim to address the limitations identified in this study. Prospective, multicenter studies with standardized surgical techniques and histopathological assessments are needed to confirm the predictive value of varicocele grade and testicular histopathology on sperm retrieval outcomes in men with NOA. Increasing the sample size and including multiple centers can enhance the statistical power and generalizability of the results, particularly for subgroups with rare histopathological patterns like EMA and SCOS.

Incorporating a control group of NOA patients with varicocele who do not undergo varicocelectomy would allow for a more definitive assessment of the surgical intervention’s effectiveness. Randomized controlled trials comparing varicocelectomy with observation or alternative treatments could provide high-level evidence to guide clinical decision-making.

Further studies should explore the long-term reproductive outcomes of patients undergoing varicocelectomy, including pregnancy rates, live birth rates, and offspring health. This information would help determine the ultimate efficacy of the procedure in achieving biological paternity and inform patient counseling.

Given the observed relapse to azoospermia in some patients, research into the mechanisms underlying transient versus sustained spermatogenesis after varicocelectomy could identify factors that predict long-term success. Investigating genetic and epigenetic factors, as well as potential adjunctive therapies to enhance or maintain spermatogenesis post-surgery, may provide new avenues for improving outcomes.

Finally, standardizing protocols for sperm cryopreservation following varicocelectomy and determining the optimal timing for sperm retrieval and storage could maximize reproductive options for patients, particularly those at risk of relapse.

## 5. Conclusions

Simultaneous microsurgical varicocelectomy and mTESE significantly enhance spermatogenesis in men with NOA, especially those with higher-grade varicoceles (Grades II and III). Varicocele grade emerges as a crucial preoperative predictor of surgical success, aiding andrologists in identifying patients most likely to benefit from these procedures. In clinical practice, varicocele grade should be utilized as a key factor when counseling NOA patients about the potential benefits of varicocelectomy and mTESE. Patients with Grade II and III varicoceles should be informed about the higher likelihood of successful sperm retrieval and the possibility of sperm returning to the ejaculate, whereas patients with Grade I varicoceles should be advised of the lower probability of success and consider alternative reproductive strategies. Emphasizing early sperm cryopreservation is essential due to the risk of relapse into azoospermia. By integrating varicocele grade into preoperative counseling, clinicians can provide personalized recommendations, set realistic expectations, and facilitate informed decision-making, ultimately optimizing patient selection for varicocelectomy and mTESE and improving fertility outcomes in men with NOA.

## Figures and Tables

**Table 1:** Baseline characteristics of patients.

Characteristic	Value
Total patients (n)	78
Mean age (years)	33.9 ± 7.6
Left-sided varicocele (%)	78 (100%)
Bilateral varicocele (%)	17 (21.8%)
Varicocele grade (%)	
- Grade I	24 (30.8%)
- Grade II	31 (39.7%)
- Grade III	23 (29.5%)
Mean FSH (IU/L)	18.2 ± 7.4
Mean LH (IU/L)	8.1 ± 2.9
Mean testosterone (ng/mL)	3.1 ± 1.4

FSH: Follicle-stimulating hormone; LH: Luteinizing hormone.

**Table 2:** Sperm return to ejaculate and testicular sperm retrieval rates by histopathological patterns.

Histopathological Patterns	Patients (n)	Patients with Sperm Detected in Biopsy (n)	Sperm Retrieval Rate in Biopsy (%)	Patients with Sperm Return to Ejaculate (n)	Sperm Return to Ejaculate Rate (%)
HYPO	33	28	84.8	22	66.7
EMA	4	0	0	0	0
LMA	17	8	47.1	4	23.5
SCOS	24	0	0	0	0

HYPO: hypospermatogenesis, EMA: early maturation arrest, LMA: late maturation arrest, SCOS: Sertoli cell-only syndrome.

**Table 3:** Multivariate logistic regression analysis predicting postoperative sperm retrieval.

Variable	Odds Ratio (OR)	95% Confidence Interval (CI)	*p*-Value
Varicocele Grade			
- Grade II vs. Grade I	5.87	1.48–23.28	0.012 *
- Grade III vs. Grade I	4.35	0.90–21.07	0.067
Histopathological Findings			
- HYPO vs. SCOS	8.25	2.06–33.02	0.003 *
- LMA vs. SCOS	7.60	1.08–53.48	0.041 *
Age (years)	1.02	0.95–1.10	0.560
FSH (IU/L)	0.98	0.91–1.05	0.610
LH (IU/L)	1.03	0.88–1.20	0.710
Testosterone (ng/mL)	1.10	0.80–1.50	0.550
Bilateral Varicocele (Yes vs. No)	1.20	0.40–3.60	0.750

HYPO: hypospermatogenesis, LMA: late maturation arrest, SCOS: Sertoli cell-only syndrome, FSH: Follicle-stimulating hormone, LH: Luteinizing hormone. Statistically significant *p*-values are indicated with an asterisk *.

## Data Availability

Data supporting the reported results are available from the corresponding author upon reasonable request.
